# Summarizing methods for estimating population size for key populations: a global scoping review for human immunodeficiency virus research

**DOI:** 10.1186/s12981-022-00434-7

**Published:** 2022-02-19

**Authors:** Chen Xu, Fengshi Jing, Ying Lu, Yuxin Ni, Joseph Tucker, Dan Wu, Yi Zhou, Jason Ong, Qingpeng Zhang, Weiming Tang

**Affiliations:** 1 Medical Record Information Section, Yantai Yuhuangding Hospital, 264000 Shandong, China; 2 Dermatology Hospital of Southern Medical University, Guangzhou, China; 3 University of North Carolina Project-China, No. 7, Lujing Road, Yuexiu District, Guangzhou, 510095 China; 4 Institute for Artificial Intelligence, Guangdong Second Provincial General Hospital, Guangzhou, China; 5 School of Data Science, City University of Hong Kong, No. 83 Tat Chee Avenue, Kowloon Tong, Kowloon, Hong Kong SAR China; 6 Faculty of Infectious and Tropical Diseases, London School of Hygiene and Tropical Medicine, London, UK; 7 Division of Infectious Disease, School of Medicine, University of North Carolina at Chapel Hill, Chapel Hill, NC USA; 8 West China School of Public Health, West China Medical Center SCU, Chengdu, China; 9 Zhuhai Center for Diseases Control and Prevention, Zhuhai, China; 10 Faculty of Medicine, Macau University of Science and Technology, Macau SAR, China; 11 Melbourne Sexual Health Centre, Alfred Health, Melbourne, Australia; 12 Central Clinical School, Faculty of Medicine, Monash University, Melbourne, Australia

**Keywords:** HIV, Key population, Population size estimation, Scoping review

## Abstract

**Background:**

Estimating the population sizes of key populations(people who inject drugs, men who have sex with men, transgender persons, and commercial sex workers) is critical for understanding the overall Human Immunodeficiency Virus burden. This scoping review aims to synthesize existing methods for population size estimation among key populations, and provide recommendations for future application of the existing methods.

**Methods:**

Relevant studies published from 1st January 2000 to 4th August 2020 and related to key population size estimation were retrieved and 120 of 688 studies were assessed. After reading the full texts, 81 studies were further excluded. Therefore, 39 studies were included in this scoping review. Estimation methods included five digital methods, one in-person method, and four hybrid methods.

**Finding:**

We summarized and organized the methods for population size estimateion into the following five categories: methods based on independent samples (including capture-recapture method and multiplier method), methods based on population counting (including Delphi method and mapping method), methods based on the official report (including workbook method), methods based on social network (including respondent-driven sampling method and network scale-up method) and methods based on data-driven technologies (Bayesian estimation method, Stochastic simulation method, and Laska, Meisner, and Siegel estimation method). Thirty-six (92%) articles were published after 2010 and 23 (59%) used multiple methods. Among the articles published after 2010, 11 in high-income countries and 28 in low-income countries. A total of 10 estimated the size of commercial sex workers, 14 focused on men who have sex with men, and 10 focused on people who inject drugs.

**Conclusions:**

There was no gold standard for population size estimation. Among 120 studies that were related to population size estimation of key populations, the most commonly used population estimation method is the multiplier method (26/120 studies). Every method has its strengths and biases. In recent years, novel methods based on data-driven technologies such as Bayesian estimation have been developed and applied in many surveys.

## Background

The global Human Immunodeficiency Virus epidemic disproportionately affects key populations, including people who inject drugs (PWID), men who have sex with men (MSM), transgender persons and commercial sex workers(CSW) [1]. Key populations are vulnerable groups of HIV infection due to specific higher-risk behaviors: PWID were chosen because of the sharing of needles and syringes; MSM were chosen because of anal sex without condoms; CSW were chosen because of the total frequency of sexual behaviors (the larger total numbers, the larger risky numbers). Understanding the HIV burden among the key populations is essential for estimating the overall burden of HIV both globally and regionally. Population size estimation is an important step towards understanding the HIV burden, and accurate size estimation of key populations can inform resource allocation and distribution of HIV prevention services. However, due to the hidden nature of some of these populations, estimating the population size of key populations is challenging. First, the methods for population size estimation have intrinsic biases. For example, data inputs used by some methods may not reflect actual conditions if the quality of data can not be promised [2, 3]. Second, key populations may be hard to reach because of various reasons, such as social stigma and discrimination [4, 5].

Existing literature related to the size estimation of the key population demonstrated the strengths and shortages of the currently existing methods [6]. However, very few studies have systematically summarized the categories of previously used methods or pointed out their problems, which did not provide further guidance in using these methods in the future study. The traditionally used methods have various intrinsic biases. Besides, the availability of reliable and authentic data has been a big challenge [7]. For example, acknowledging the existence of key populations by public health facilities or the government is challenging [8]. Estimating the size of the key populations is particularly challenging in Eastern Mediterranean, Middle East, and North Africa Region because conservative social and religious values may cause harsh judgment and may even bring life-threatening punishment [9].

There are several papers comparing different population size estimation methods, though usually restricted in specific area or limited method categories [10–12]. However, how to find the best strategy based on the local context is the current knowledge gap. To fill the knowledge gap, this scoping review examined population size estimation methods in different settings among key populations. This study aimed to summarize the application of the existing population estimation methods and discuss their respective strengths and weaknesses.

## Methods

### Search strategy

Relevant studies published from January 2000 to 4th August 2020 and related to population size estimation were retrieved from PubMed [13]. Search terms were chosen based on the relevance to the topic of this study. Search terms included "people who inject drugs"; "men who have sex with men"; "transgender persons"; "sex workers" in combination with: "size estimate" and "size estimation". We used the PRISMA checklist for scoping reviews. This review was completed on 20th August 2021.

### Selection criteria

After de-duplication, the nonduplicate publications were retrieved from PubMed, and further reviewed independently by two researchers to determine to identify the final studies to be included. Only publications related to the sampling methods of population size estimation among the key populations and have referential meaning for the application of these various methods were included in the final review. We excluded studies that were not related to the topic of this review or had no suggestive meaning for the future design of population size estimation methods. The titles, abstracts, and full texts of all publications were screened by two independent reviewers (FJ and CX). If it was not clear whether a study should be included in the final review, the three authors (FJ, CX, and WT) reviewed the full texts together to discuss whether the article met the inclusion criteria.

### Data extraction

A standardized extraction form was performed using Microsoft Excel to extract the first author, date of publication, and size estimation sampling method of key populations. The publications were categorized into five categories. These include methods based on independent samples, methods based on population counting, methods based on the official report, methods based on social networks, and methods based on data-driven technologies.

### Text mining

Text mining, also named text data mining, refers to the process that adopts computer science and artificial intelligence technologies in natural language processing tasks for extracting structured information from unstructured text. Through text mining, we can identify meaningful patterns and new insights. In order to illustrate research trends of HIV key population size estimation papers, we employed a semantic analysis tool, CiteSpace, which is particularly commonly used in the discipline of scientometrics. Text mining results based on full text of all selected studies, this tool can help us to develop relation graphs of important research words in structured items. Notice that Citespace can only run on the platform of Web of Science, then thus our full text mining results are based on studies whose full text could be retrieved on Web of Science (i.e., all eligible full-text studies). Furthermore, this tool can also display relations among key words of existing research. In summary, to develop relation graphs among keywords as well as research trends about the topic of HIV key population size estimation, we utilized text mining of all eligible full-text studies to better capture the relationships among several keywords.

## Results

**Fig. 1:**
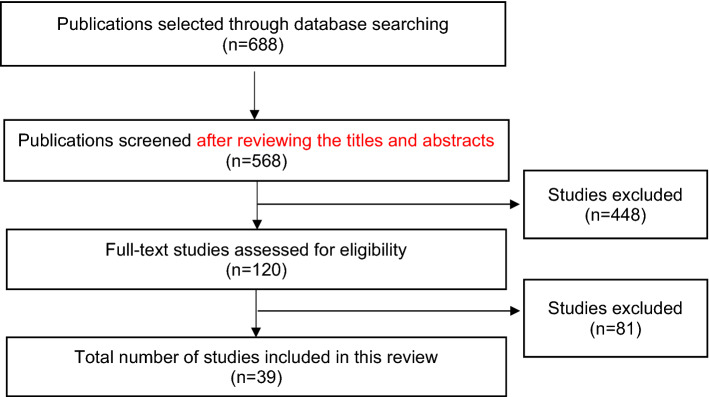
Flowchart of the review

Overall, 688 citations were retrieved from the initial search. After reviewing the titles and abstracts, 568 manuscripts that were not relevant to the topic of this paper were excluded, leaving 120 full-text manuscripts. After reading the full texts, 81 studies were further excluded. Therefore, 39 studies were included in this scoping review (Fig. 1).

### Findings

Among the included studies, seven used capture-recapture method, six used multiplier method, two used Delphi method, three used mapping methods, three used workbook method, six used network scale-up method, six used RDS method, three used Bayesian estimation method, two used Laska, Meisner, and Siegel (LMS) estimation method [14] and one used stochastic simulation method (Appendix 2). Among the articles reviewed, 36 (92%) articles were published after 2010 and 3 (8%) were published before 2010. Sixteen (41%) studies examined one method and 23 (59%) studies used multiple methods. 11 studies set the research context in high-income countries and 28 in low- and middle-income countries. A total of 10 estimated the size of commercial sex workers, 14 focused on MSM, and 10 focused on people who injected drugs (PWIDs). These population estimation methods included five digital methods, one in-person method, and four hybrid methods. Appendix 1 summarizes the publications included in this review.

**Fig. 2:**
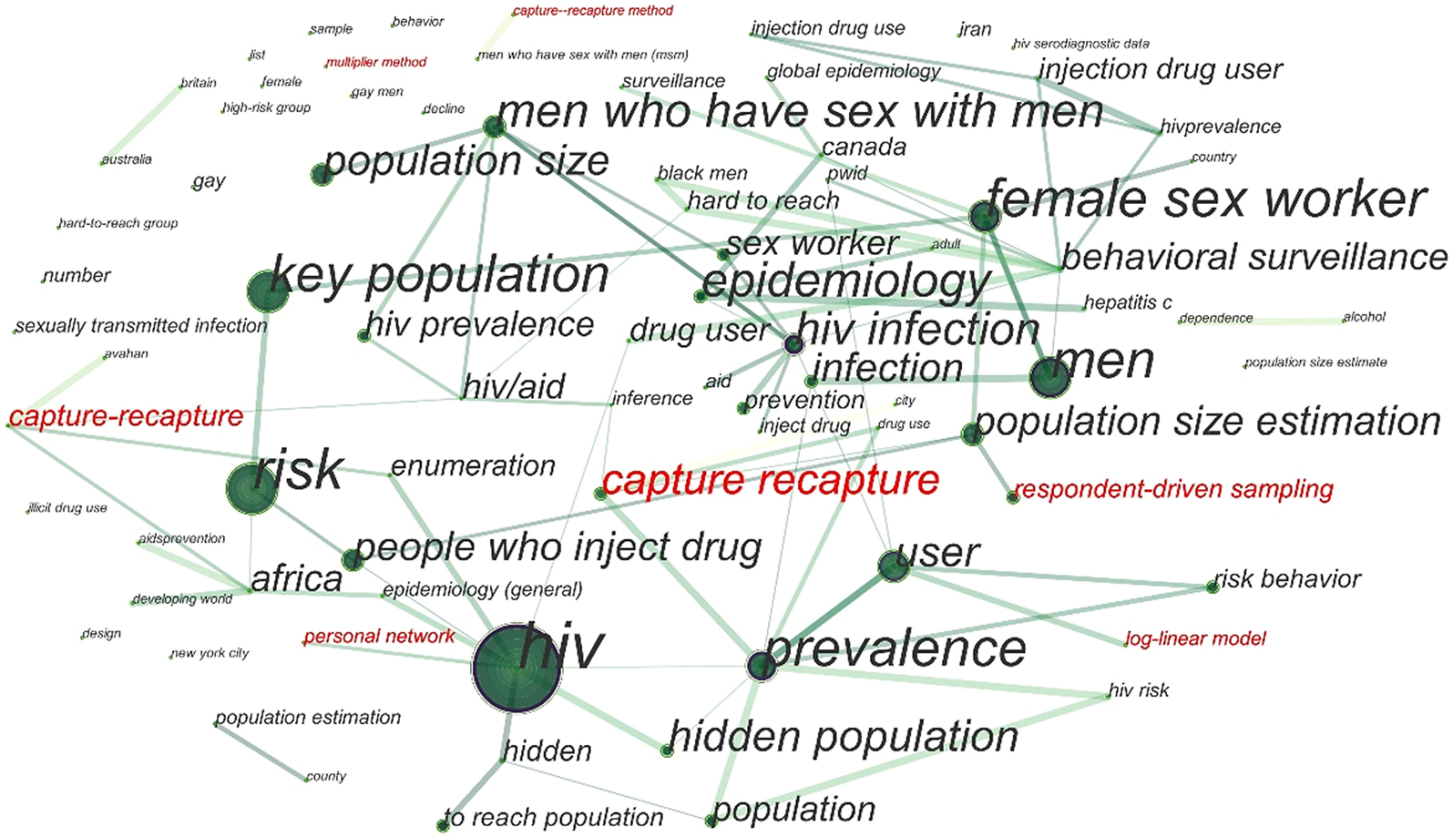
Relation graph of important words from full-text mining

**Fig. 3:**
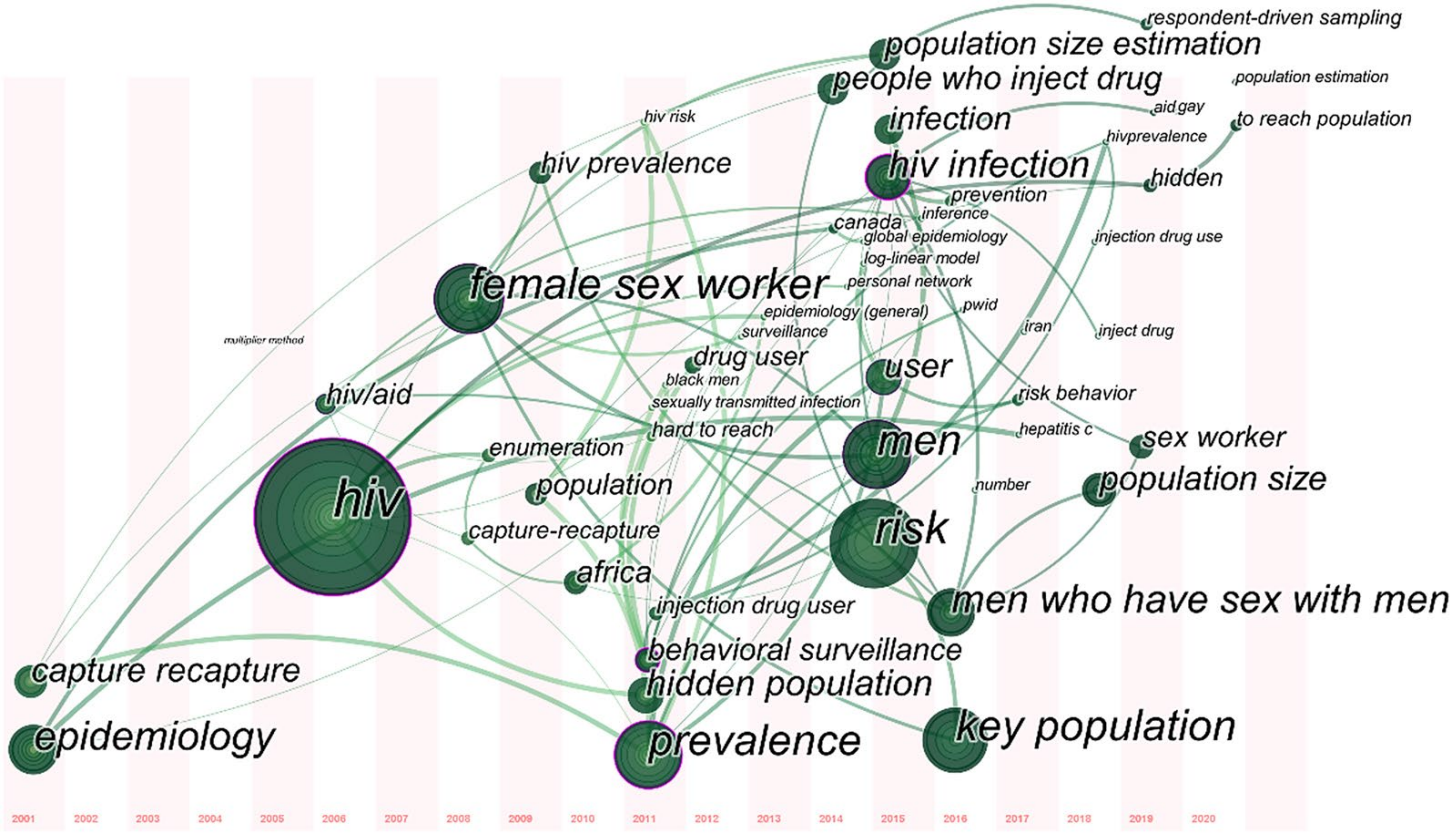
Research trends of important words from 2001 to 2020 from full-text mining

Legend: This graph shows the relations among different keywords from full-text mining. The red font ones are important items relevant to size estimation methods, which are the research objective in our paper (because we do not study on items such as "Africa" and "risk behavior" in our study, hence they are labeled in black font). The appearing times and word size of each item can show its importance and relation centrality in this topic of research (i.e. size estimation for HIV key populations).

We used full-text mining of 120 full-text articles that could be retrieved on the Web of Science. Figure 2 shows relationships among several research key points including reference citing and semantic understanding. The capture-recapture method appeared three times in this graph with several edges. Social network-based methods such as RDS and network scale-up (i.e., calling 'personal network' from full-text semantic extraction) were also in relatively big word size in this knowledge graph which represents the frequency of mentions. It should be noted that the key item named 'log-linear model' is relevant to Bayesian estimation and LMS estimation. Other methods like Delphi and the workbook method are more likely to be independent as they are even not shown up in this knowledge graph. Figure 3 represents the research trend of this topic in the preceding 20 years. We observe that the methods used gradually changed from traditional methods (e.g., capture-recapture) to social network-based ways (e.g., RDS).

**Fig. 4:**
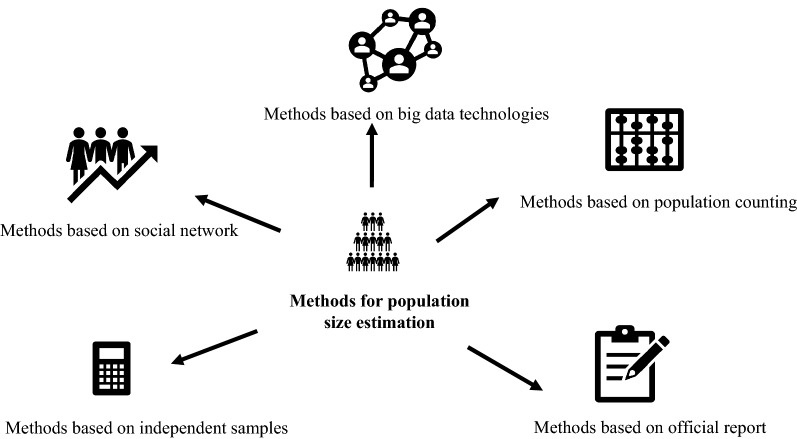
Type of methods for population size estimation

We summarize the methods for population size estimation and categorized them into the following five categories (Fig. 4): methods based on independent samples, methods based on population counting, methods based on the official report, methods based on the social network, and methods based on data-driven technologies. Table 1 represents the summary of 10 commonly used population size estimation methods.

**Table 1:** Summary of size estimation methods. The continuity of this table is across four pages

Sampling method	Description	Assumption	Strength	Weakness
Capture-recapture [15]	Assesses the overlaps between incomplete case lists from multiple independent data sets	1) the selected sampled population is a good representation of the whole population 2) the sample is a closed population 3) able to match individuals in both datasets; 4) individuals have an equal likelihood of being captured	Simple and easy to use for researchers	Capture biases: not everyone has an equal chance of being captured; Estimates would be too high if matches were not identified or too low if recaptures were matched incorrectly
Multiplier [16]	Two independent sources of data are used to make the estimation, including an authentic count or list of the population whose size is being estimated and a survey of the populations whose size is being estimated	There is accurate demographic and geographic information of the key population	Simple and easy to use	The quality of the data can cause bias; the resulting survey samples may not be fully representative of the key population
Delphi [17]	Estimating the size of key populations by the individual judgment of several experts	The estimation from an expert team could accurately reflect the reality	Low cost with high efficiency	The estimation may be subjective and not reliable because of the quality of the expert team; Lack of strategies to deal with the disparity between the experts
Mapping [18]	The locations of the key population are systematically identified and mapped to estimate the size of the key population	The quality of the data can be guaranteed by the full involvement of the key populations	The estimate is made with transparency	The missing of some geographical locations may underestimate the size of key populations; overestimation may happen if the key population frequently attend multiple locations
Workbook [19]	The key population is identified first and then the estimates are combined with the total population to calculate the proportion of the key population in a specific region	Typically used in countries or regions where the epidemic is low and concentrated	The estimate is made with transparency; errors can be prevented by automatic consistency and audit check	In some countries, data may be limited because of stigma and discrimination among the key populations and legal issues, which may make data unreliable or of poor quality
Network scale-up [20]	Respondents are asked about the behaviors of acquaintances from their social network to estimate the number of key populations from the social network of each respondent	The average size of personal networks of key populations and the population as a whole are the same; People can accurately report the behaviors of acquaintances from their social networks	The privacy of the key populations is protected because the researchers do not directly contact them	The respondents may ignore key populations among their acquaintances (transmission error); Obtaining a representative sample is challenging because of stigma and discrimination
Respondent Driven Sampling [21]	A sample from the key population is selected purposively and then these selected individuals are given coupons to recruit other key populations from their social network	Recruiters randomly pass coupons to their social network members who are members of the key populations; Every participant has only one chance to receive the coupon and is equally likely to be recruited;	The Respondent-Driven Sampling method is an effective sampling method for estimating hard-to-reach networked populations with no sampling frames	Limited recruitment within the key populations may lead to biased estimates
Bayesian Estimation [22]	The key population size is estimated following Bayes' theorem, which is based on a prior probability distribution	If there exists some prior knowledge, like prior probability, the Bayesian method is suitable	It can solve the problem when there is no direct data to estimate the population size for a specified geographical area through survey sampling studies by utilizing empirical data	Bayesian methods might be subjective, due to different researchers with different prior beliefs
Stochastic Simulation [23]	Estimating the size of a certain population (e.g., HIV-positive) using epidemiologic data using the Monte Carlo method	Parameters are based on the data from representative clinical trials or observational cohort studies	Stochastic simulation makes it possible to naturally produce plausibility intervals for estimates in the face of uncertainty	First, some complex simulation process is quite time-consuming. Second, thanks to different kinds of parameters setting and the unknown quality of observed data, the robustness of some simulation model estimates is not stable
Laska-Meisner-Siegel Estimation [24]	Based on a single sample and in a single venue, it is an unbiased estimator for the size of a population	This method assumes that we only have a one-time sampling	This estimation method is time- and resources- saving, when comparing with capture-recapture	This method only requires one single sample, thus its estimation accuracy might be lower than other several times sampling estimation methods

## Methods based on independent samples

### Capture-recapture

Although some novel methods for population size estimation have emerged in recent years, a large number of surveys have been conducted using the capture-recapture method. This method can provide accurate estimates at a low cost [12, 25]. In general, the premise of this analysis is based on the overlap between several samples of the key population [26]. The process of the capture-recapture method includes two separate captures [27]. Key populations are marked and counted in the two captures independently. Some participants captured in the second capture may have already been marked in the first capture. In order to prevent the collection of personal identification information, unique objects such as coupons are commonly used to identify recaptures. However, calculating the number of recaptures is challenging because the databases used may not record the same unique objects from individuals [15]. In some cases, there is no way to determine if the person with the unique object in the second capture is the same person who received it in the first capture [28]. Bias may exist because on some occasions key populations would surround the researcher who is distributing the objects because they hope to get the object. The choice of an appropriate unique object and distributors are of vital importance to guarantee a successful capture-recapture sampling [29, 30]. This approach is highly adaptable for key populations such as drug users and commercial sex workers. It is recommended for use when a census or good-quality data are not available.

### Multiplier

The multiplier method is always integrated with other methods, such as the respondent-driven sampling method to estimate the size of the key populations. There were three different types of multipliers among the publications reviewed, including service multiplier, unique object multiplier, and web/mobile Apps multiplier [10]. The service multiplier method uses the programmatic data collected from key populations by health centers [31]. The unique object multiplier method refers to randomly distributing the unique object to the key populations [12]. The web/mobile Apps multiplier method assessed the use of a certain website or mobile phone application among the key populations [32]. The accuracy of the multiplier is highly dependent on the quality of the data source [11]. In addition, different data sources can produce different estimations [33]. To improve the reliability and validity of the multiplier, the representativeness of the data source and the completeness of the benchmark should be considered in advance when conducting the survey.

## Methods based on population counting

### Delphi

The Delphi method refers to convening a group of experts to synthesize and interpret the information in order to estimate population size [17]. Typically, this method acts as a way to reach an agreement about the estimates from other methods. The team of experts usually consists of those who are familiar with the local geography and culture from local government, research institutions, and social community sectors. Generally, the median, upper and lower limit for the estimate are identified based on local and international data and the expert opinion of the Delphi panel [31]. Experts' opinions will be gathered with discussion to reach a consensus that represents the "best" estimates. This method is vulnerable to subjectivity. Bias may arise when the expert team has a limited understanding of the demographic or geographic features of the populations whose size is being estimated.

### Mapping

Mapping is similar to the cross-sectional study in epidemiological research. This method identifies the sites where key populations gather, such as public spaces, mobile apps, and websites. Using map sites to estimate the number of populations at each site begins with identifying locations frequented by key populations [18]. Only the sites mostly frequented by key populations are identified and reported. Mapping relies on the numeric estimates of key informants instead of the count of key populations at each identified site, thus there may be differences between different respondents interviewed at various sites [34]. The variability of the estimates of different respondents could influence the accuracy of the overall estimation [35]. Overestimating or underestimating the number of key populations may happen. The participation of the key populations depends on the extent of their visibility so some individuals may have been omitted, which will lead to underestimation. This method could also overestimate the number of key populations if they frequent multiple locations.

## Methods based on the official report

### Workbook method

The workbook method uses data retrieved from health officials at the national or regional level [19]. It relies on the existing official records [36]. This method emphasizes a range of estimates instead of a single point estimate. The workbook method uses regional spreadsheets to make estimations of various areas. The data are from the surveillance system and large-scale screening to gain an understanding of the distribution of the key populations [37]. Inevitably, some regions may not have available data to make an estimation. Missing data are estimated by the data from the area with the most similar socioeconomic and geographic features. In addition, the estimation of missing data is usually adjusted by health officials and experts who are familiar with the area.

## Methods based on social network

### Network scale-up

A network scale-up method is a promising approach to population size estimation. This method starts with estimating the size of a personal network in a small sample. The size of the network of each individual is estimated by predicting the number of key populations they know instead of asking questions about their behaviors directly [38]. This follows estimating the number of people of key populations among the total population. The major assumption of this method is that the social network of individuals involved in the survey can represent the total population [20]. The average personal network size in a certain area can be calculated by averaging the individual value of reported key populations over a large number of respondents [39]. Each individual’s report of their network contributes to the estimation. The main challenge of this method is to determine the sample size required since no individual has complete knowledge about all their acquaintances [40]. The strength of the network scale-up method is that it does not require access to key populations except for people from the initial random sample. The main bias of this method is that estimating the size of a personal network can be cognitively demanding [41]. Different people may have different definitions of key populations and acquaintances [42].

### Respondent-driven sampling

Respondent-driven sampling method is increasingly prevalent for population size estimation of key populations in recent years [43]. Many publications have demonstrated the success of peer-driven recruitment in collecting data for key populations. It is a network-based sampling method that starts from recruiting a selected sample from the key populations and respondents recruit their peers from their networks [44]. The purposively selected sample is named "seeds", who recruit other members [21]. There is always a limit for recruitment, usually 3–5 people [45]. Coupons, quotas, and incentives are used to assist the recruitment. The coupons are given from the "seeds" and then passed to other members of the key population [46]. The financial compensation for the participation of the key population could facilitate the development of the recruitment chain. Each recruitee could potentially become a recruiter, which makes the recruitment continue in waves [47]. The connection between recruiters and recruiters can then be traced using the unique identification of coupons. The longer the chains of recruitment, the more representative the surveyed sample [48]. Even though longer recruitment chains could reduce potential selection bias, there are still chances for bias. For example, some populations whose activity is stigmatized may decline participation. In addition, the quality of RDS highly depends on the number of seeds used at the beginning of the study [49].

## Methods based on data-driven technologies

### Bayesian estimation

The Bayesian estimation method is based on a prior probability distribution using Bayes' theorem to estimate the new probability. The Bayesian estimation assumes that prior probabilities can be used to enhance estimation [22]. If the countries or cities are areas with no direct data on such population size, and there exists a prior probability, the Bayesian estimation method is well suitable [50]. However, different investigators may have a different understanding of prior knowledge according to everyone's subjective realization. As a result, they might give different prior distributions and then obtain different posterior distributions, resulting in the subjectivity of this method.

### Stochastic simulation

The stochastic simulation model is to estimate a population-based on epidemiologic data. Stochastic simulation (Monte Carlo) firstly generates a simulated system and then analyze it through probability models based on limited observed data [23]. When we have information from observational cohort studies and clinical trials, such data can help to set simulation parameters, and then simulation models may work. When we have rich epidemiologic data, we can use stochastic simulation models to estimate population size. The strength of this method lies in the ability to produce plausibility ranges for estimates, which describe the uncertainty surrounding the estimates, based on the data to which the model was calibrated [51]. As for shortcomings, first, some large-scale complex simulation processes can be time-consuming. Second, the validity of model estimates is highly dependent on the quality of available data used to calibrate the model.

### LMS estimation

Laska, Meisner, and Siegel developed an unbiased estimator for the size of a population in a single venue based on a single sample [14]. Laska, Meisner, and Siegel estimation for MSM size population is based on one single sampling [24]. In other words, this method assumes that we only have a one-time sampling. Compared with other population size estimation methods, first, compared with the capture-recapture method, this method only needs one single-time "capture", hence it is time-saving and resource-saving. Second, when comparing with the multiplier method, it is more scientific according to some statistical principles. However, in the field of statistics, this method is quite traditional and a little hard to make some huge contributions or incorporate some novel revisions [52]. However, as this method only requires one single sample, thus its estimation accuracy might be lower than other population size estimation methods.

## Issues of existing population size estimation

Data accuracy, the skills of investigators, duration of size estimation studies, the involvement of the community, geographical areas, and costs and resources required for population size estimation are all essential factors to influence the accuracy of the size estimation result [7].

The current size estimation methods have several limitations. First, further evaluation of the impact of the potential bias and how the biases may impact the size estimation of the key population is needed. Second, it is still hard to take the hardest to reach individuals into consideration. Traditional methods such as capture-recapture and the multiplier method extract independent samples from the population. It is challenging to achieve when the populations are hidden. Social stigma also makes accurate estimation of the size of key populations challenging. In addition, the engagement of people with illegal behaviors to disclose their behaviors or social network to interviewers may cause serious bias. Considering selling sex is legal in some countries but not in many other countries, this is closely related to local contexts.

**Fig. 5:**
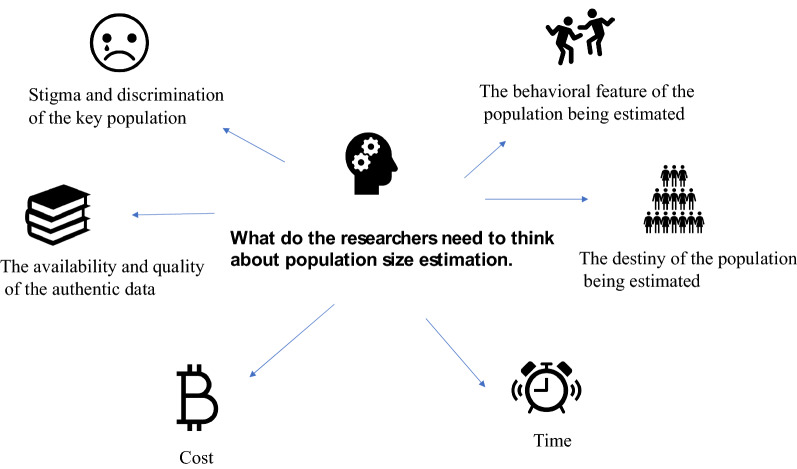
The researchers need to think about when choosing methods for population size estimation

We summarize things that the researchers need to think about when choosing methods for population size estimation into the following six categories (Fig. 5). Results may vary for the same population by using different methods. For example, when estimating the population size of MSM, using the capture-recapture method may overestimate the actual number of the population because the mobility of the population being estimated makes the number of recapture population decrease. Using the Multiplier method may not get the actual number of the population because it highly depends on the quality of the data source. In addition, the result may be underestimated because the population being estimated is hard to reach. Delphi method is vulnerable to the subjectivity of the expert team, especially when experts have limited understanding of the demographic or geographic features of the populations whose size is being estimated. Using the network scale-up method may underestimate the size of the population being estimated because the respondents may not have complete knowledge about all their acquaintances, which means the estimation can be cognitively demanding.

## Discussions

This scoping review has several implications. Developing improved methods to measure the size of populations of the key population is demanding. We need a novel, comprehensive method for population size estimation that avoids the aforementioned challenges. Use different methods to fill the limitation of the estimation methods and to balance the strengths and weaknesses of the used method would be critical to deriving the final estimate.

First, when choosing the method for population estimation, we should consider the potential bias associated with each approach. For example, traditional social network-based methods are collecting data from the MSM population all the time, which might cause some potential bias called convenient sample bias.

Second, for the selection of the methods, we need to tailor this based on the features of the key population, local context, and costs. Evidence from a meta-analysis of multiple sources and Delphi panels could be applied where several findings have been performed on the population whose size is being estimated [53]. Behavioral surveys among the key populations should be conducted before the survey. Planning and preparation will improve the validity of the estimates. If possible, working with members from the key population whose size is being estimated in the community may help better select the most appropriate methods. A pilot study among the subsample of the population whose size is being estimated is a valid approach.

Third, using advances in technology and data science to assist the estimation might be the future trend. As mentioned before, from Fig. 3, we can know that the research trend of utilized methods of this topic in recent 20 years is gradually changing from traditional ways like capture-recapture to social network-based ways like respondent-driven sampling. Perhaps that means the social network data could have great potential in developing accurate estimation models. With the rapid development of data-driven technologies, novel machine learning methods like graph convolutional networks [54] and generative adversarial networks [55] have become popular in Artificial Intelligence (AI) field. Using these new data-driven methods in size estimation tasks for public health research might be a valuable try in the future. Furthermore, using data-driven technologies correctly could be friendly to key populations, because such data-driven approaches depend on existing accessible non-sensitive data, as other model-driven estimation methods may require some hard-reachable data which are private.

## Conclusions

The population size estimation methods continue to have limitations. Different methods are likely to give very different results. The estimates depend on subjective judgments, the quality of authentic data and assumptions are always hard to meet.

## Data Availability

The datasets used and/or analysed during the current study are available from the corresponding author on reasonable request.

## Appendix 1

See Table 2.

**Table 2.** Characteristics of individual studies included in the scoping review.

No.	ID	Estimation method	Key populations	Study settings
1	E B Hook et al.	Capture-recapture	The key populations	The United States
2	M C Buster et al.	Capture-recapture	PWID	Amsterdam
3	Ruiz MS et al.	Capture-recapture	PWID	Washington DC
4	Apodaca K et al.	Capture-recapture	MSM	Uganda
5	Karami M et al.	Capture-recapture	CSW	Tehran, Iran
6	Doshi RH et al.	Capture-recapture	PWID, MSM, and CSW	Kampala, Uganda
7	Li G et al.	Capture-recapture	MSM	Beijing, China
8	Sulaberidze L et al.	Multiplier	MSM	Tbilisi, Georgia
9	Okal J et al.	Multiplier	PWID, MSM, and CSW	Nairobi, Kenya
10	Paz-Bailey G et al.	Multiplier	MSM and CSW	El Salvador
11	Burrell ER et al.	Multiplier	MSM	the United States
12	Rich AJ et al.	Multiplier	MSM	Metro Vancouver, Canada
13	Hiebert L et al.	Multiplier	PWID	Malaysia
14	Khalid FJ et al.	Delphi	PWID, MSM, and CSW	Unguja Island, Zanzibar
15	Okal J et al.	Delphi	PWID, MSM and CSW	Nairobi, Kenya
16	Bunjaku DG et al.	Mapping	PWID, MSM and CSW	Kosovo
17	Odek WO et al.	Mapping	CSW	Kenya
18	Wambura M et al.	Mapping	MSM and CSW	Tanzania
19	Lu F, Wang N et al.	Workbook	The key populations	China
20	Ha NTT et al.	Workbook	PWID	Son La, Vietnam
21	Lansky A et al.	Workbook	heterosexual persons	the United States
22	Scholz SM et al.	Network scale-up	MSM	Germany
23	Baral S et al.	Network scale-up	MSM	Multiple countries
24	Guo J et al.	Network scale-up	MSM	Beijing, China
25	Ezoe S et al.	Network scale-up	MSM	Japan
26	Maghsoudi A et al.	Network scale-up	PWID and CSW	Iran
27	Wang J et al.	Network scale-up	MSM	Shanghai, China
28	Bengtsson L et al.	Respondent-driven sampling	MSM	Vietnam
29	Holland CE et al.	Respondent-driven sampling	MSM and CSW	Burkina Faso and Togo
30	Johnston LG et al.	Respondent-driven sampling	males who inject drugs	Myanmar
31	Carballo-Diéguez A et al.	Respondent-driven sampling	MSM	Buenos Aires
32	Buchanan R et al.	Respondent-driven sampling	PWID	Multiple countries
33	Lachowsky NJ et al.	Respondent-driven sampling	MSM	Vancouver, Canada
34	Overall AM et al.	Bayesian estimation	PWID	Scotland
35	Datta A et al.	Bayesian estimation	MSM	Côte d'Ivoire
36	Bao L et al.	Bayesian estimation	PWID	Bangladesh
37	Nakagawa F et al.	Stochastic simulation	HIV-positive MSM	UK
38	Chen H (2013) et al.	Laska-Meisner-Siegel estimation	MSM	Changsha, China
39	Chen H (2011) et al.	Laska-Meisner-Siegel estimation	MSM	One city in China

## Appendix 2

See Table 3.

**Table 3:** The summary of population size estimation methods categories

Categories	Definition of categories	Methods
Methods based on independent samples	Multiple independent sources of data are used to make the estimation	Capture-recapture method
		Multiplier method
Methods based on population counting	Traditional geolocation and counting measures are used to estimate the size of the key population	Delphi method
		Mapping method
Methods based on the official report	The estimates are made by combining them with the official report	Workbook method
Methods based on social network	The key population is recruited from their social network	Respondent-driven sampling method
		Network scale-up method
Methods based on data-driven technologies	The key population size is estimated by data-driven technologies	Bayesian estimation method
		Stochastic simulation method
		LMS estimation method

## Abbreviations

MSM: Men who have sex with men
PWID: People who inject drugs
CSW: Commercial sex workers
HIV: Human Immunodeficiency Virus
RDS: Respondent driven sampling
LMS: Laska, Meisner, and Siegel
UK: The United Kingdom
